# Improving Respiratory Support Practices to Reduce Chronic Lung Disease in Premature Infants

**DOI:** 10.1097/pq9.0000000000000193

**Published:** 2019-08-09

**Authors:** Bernadette M. Levesque, Laura Burnham, Natasha Cardoza, Marsha Adams, Robyn Cohen, Mark Mirochnick, Alan Fujii, Bharati Sinha

**Affiliations:** From the * Division of Neonatology, Boston Medical Center, Boston, Mass.; † Boston University School of Medicine, Boston, Mass.; ‡ Division of General Pediatrics, Boston Medical Center, Boston, Mass.; § Department of Respiratory Therapy, Boston Medical Center, Boston, Mass.; ¶ Department of Nursing, Boston Medical Center, Boston, Mass.; ‖ Division of Pediatric Pulmonology, Boston Medical Center, Boston, Mass.; ** Department of Pediatric Newborn Medicine, Brigham and Women’s Hospital, Boston, Mass; †† Harvard Medical School, Boston, Mass.

## Abstract

**Introduction:**

We implemented a bundle of respiratory care practices and optimized delivery of continuous positive airway pressure (CPAP) to reduce the incidence of chronic lung disease (CLD) among very low birth weight (VLBW) infants born before 33 weeks gestation.

**Methods:**

Our multidisciplinary task force utilized 6 plan-do-study-act cycles to test our interventions. The primary outcome was the quarterly percentage of infants diagnosed with CLD; other outcomes included the percentage of infants initially managed with CPAP, intubation <72 hours of age, use of a nasal cannula, and days of ventilation, oxygen, and/or CPAP. Process measures included compliance with each of the 5 components of the bundle; balancing measures included mortality and complications of prematurity.

**Results:**

Demographics were similar in the 55 infants born before and 76 infants born after the task force interventions, except for gestational age, which was lower before. CLD decreased by 55.5% (from 37.5% to 16.7%). Quarterly percentage of infants requiring intubation decreased from 87.5% to 40.8%. Quarterly average days of ventilation decreased from 11.2 to 6.1, and days of supplemental oxygen declined from 44.1 to 25.4, while the use of CPAP increased. There were no differences in adverse events including mortality, pneumothorax, use of postnatal steroids, or any retinopathy of prematurity. The incidence of patent ductus arteriosus declined from 60% to 33% (*P* < 0.01).

**Conclusions:**

We reduced the incidence of CLD among our very low birth weight infants born before 33 weeks gestation by over 50% without increasing any measured adverse outcomes. The incidence of patent ductus arteriosus declined.

## Abstract 2

Supplemental Digital Content is available in the text.

## INTRODUCTION

Very low birth weight (VLBW) infants born before 33 weeks gestation at Boston Medical Center (BMC) had a high incidence of chronic lung disease (CLD), averaging 35% from 2006 to 2012. Several efforts to reduce the incidence of CLD were unsuccessful.

CLD is a multifactorial developmental disease, but there is a reason to believe that decreasing mechanical ventilation (MV) could reduce the incidence of CLD. In 1987, Avery et al^1^ reported that Columbia University in New York had the lowest incidence of CLD compared with 7 other major medical centers. They attributed this to early intervention with bubble continuous positive airway pressure (bCPAP) and avoidance of MV.^1^ Van Marter confirmed this finding in 2000.^2^ Since then, there have been 3 large randomized controlled trials^3–5^ and several cohort and observational studies^6–10^ that support this (or a similar) approach. All demonstrated a reduction in MV, and 3 reported a significant reduction in CLD.^6,11,12^

In 2007, a multidisciplinary team at St. Elizabeth’s Medical Center (SEMC) in Boston implemented a bundle of respiratory practices based largely upon the “Columbia” approach, including the exclusive use of bCPAP, provision of bCPAP in the DR, strict intubation and extubation criteria, and prolonged bCPAP to avoid supplemental oxygen (sO_2_) and promote lung growth.^13^ They found these practices reduced MV, and need for surfactant administration and sO_2_ among infants born at younger than 33 weeks gestational age (GA) without adverse consequences. The incidence of CLD declined by 53%.

We hypothesized that we could reduce the incidence of CLD at BMC using the same approach. However, there were significant differences between the neonatal intensive care unit (NICU) at SEMC compared with that at BMC (see **Supplemental Digital Content** at http://links.lww.com/PQ9/A114 for Supplemental Table 1), raising uncertainty about the outcome. The specific aim of this project was to reduce the incidence of CLD among VLBW infants born at younger than 33 weeks GA by 50% between the baseline period (January 1, 2012–June 30, 2013) and the intervention period (July 1, 2013–December 31, 2015) by implementing specific respiratory care practices already shown to be effective at other institutions.

## METHODS

### Context

The target population included VLBW infants born younger than 33 weeks GA; we excluded patients who died or who were transferred younger than 36 weeks postconceptual age (PCA) and not readmitted. BMC is an urban, academic, safety-net hospital that combines teaching with a mission to serve vulnerable populations; we serve a large number of uninsured, Medicaid, and low-income patients. We did not have formal respiratory care guidelines before the initiative, but Table 1 outlines representative clinical practices before and after, for comparison.

**Table 1.**
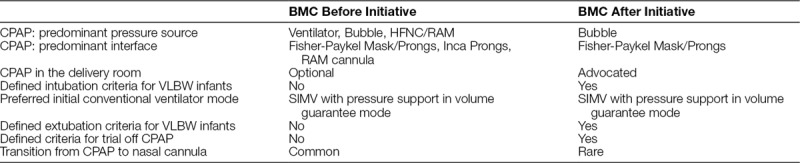
Comparison of Clinical Practices at Boston Medical Center Before and After the CLD Taskforce Initiative

### Interventions

The CLD task force team was composed of neonatologists, pulmonologists, staff nurses, and respiratory therapists (RT), and a nurse practitioner, nurse educator, and data manager. We implemented a respiratory care bundle spanning management from birth to 34 weeks PCA and optimizing continuous positive airway pressure (CPAP) delivery. The bundle included: a change in preferred mode of noninvasive support from a variety of modes to only bCPAP delivered by prongs and/or nasal mask; provision of bCPAP in the DR; establishment of intubation and extubation criteria; and use of bCPAP until infants can wean to no support/room air (RA) or until 34 weeks PCA, whichever comes first (see **Supplemental Digital Content** at http://links.lww.com/PQ9/A116 for Supplemental Figure 1). One author (B.M.L.) reviewed the rationale for these changes in a prior publication.^13^ The neonatology faculty, nursing, and RT leadership approved the bundle.

To optimize CPAP delivery we: (1) retrained the entire staff on the use of our bCPAP interface; (2) limited the use of alternate CPAP interfaces (such as nasal cannula); (3) changed from nasogastric to orogastric tubes for gastric decompression while on bCPAP; (4) introduced the use of chin straps to prevent pressure loss through the mouth while on bCPAP; and (5) encouraged frequent assessments of bCPAP interface fit and function.

### Measures

This project was a prospective longitudinal quality improvement (QI) effort based on the Model for Improvement approach that utilized plan-do-study-act cycles, evaluated interventions using process, outcome and balancing measures, and monitored data over time. The primary outcome was the quarterly percentage of infants diagnosed with CLD, defined as the need for sO_2_ at 36 weeks PCA. Other outcome measures included (1) initial management with CPAP; (2) initial CPAP success; (3) intubation <72 hours after birth; (4) use of bCPAP with nasal prongs or mask as only CPAP mode; and (5) use of nasal cannula at younger than 34 weeks PCA. We also calculated the quarterly number of days infants received CPAP, MV, or sO_2_. For the subgroup of infants born younger than 28 weeks GA, we used semiannual rather than quarterly data, due to smaller numbers. Process measures included compliance with each of the 5 elements of respiratory care, measured as the percentage of infants born during the intervention period who were managed according to the guidelines, when applicable. The tool we used to assess compliance is included as **Supplemental Digital Content** at http://links.lww.com/PQ9/A120.

Balancing measures included pneumothorax (defined by extrapleural air seen on chest radiograph); patent ductus arteriosus (PDA, defined as the presence of left to right or bidirectional ductal shunt on echocardiogram or systolic murmur with at least 2 of the following: hyperdynamic precordium, bounding pulses, wide pulse pressure, and/or pulmonary vascular congestion/cardiomegaly); any retinopathy of prematurity (ROP, defined as any stage of ROP in either eye at any time); and use of any postnatal steroids for CLD. We defined CPAP success as avoiding intubation <72 hours age and extubation success as avoiding re-intubation within 72 hours of extubation. We approximated cost only by the total length of stay.

We monitored real-time compliance with respiratory care practices and addressed deviations with neonatologists via one-on-one conversations. We provided the staff with periodic summary data regarding compliance and progress and reviewed feedback from staff during monthly CLD task force meetings.

### Analysis

We used our own center’s patient-level VON data that we downloaded into a spreadsheet and supplemented this with data specifically collected for this QI initiative. The Institutional Review Board at BMC waived the requirement for informed consent for data collection. VON permitted us to report comparative VON data and to use our own center’s patient-level data for this project.

For pre- and post-task force demographic characteristics and all measures, we used Student’s *t*-test for comparison of independent means and the *z*-test for comparison of population proportions. For all data over time, we analyzed data by run charts created with QI-Charts Version 2.0.22 Add-in for Excel, using rules for special causes to determine statistical significance.^14–16^

Run charts and median lines were created using the 16 available data points (quarterly data for 4 years). We assessed our data using the 4 rules for run charts (shift, trend, runs, and astronomical data point), but only detected shifts. For a “shift,” we calculated the number of data points above or below the initial median that would constitute a significant change using the formula log2(n)+3, where the n = number of total data points. By definition, 95% of the longest consecutive series of data points above or below the median would be expected to be within this number and data series exceeding this number would be considered significant at *P* < 0.05. With 16 data points, the number for a significant “shift” in our data is 7. When a significant change in the data was confirmed, we created new median lines for data before and after the change.

## RESULTS

A total of 149 VLBW infants with GA younger than 33 weeks were born at BMC from 01/01/2012–12/31/2015; 61 were born before our interventions, and 88 were born after. Of the 61 born before, 3 died, and 3 were transferred and not re-admitted before 36 weeks PCA. Of the 88 born after our interventions, 8 died, and 4 were transferred and not re-admitted before 36 weeks. A total of 18 infants were thus excluded from the final analysis, leaving 131 infants in our cohort, 55 before and 76 after our interventions. There were no differences in mortality or transfer rates before versus after our interventions (*P* = 0.337 and *P* = 0.912, respectively). Demographic characteristics were similar in the before and after groups except for GA, which was ~1 week lower in the before group (*P* = 0.03, see **Supplemental Digital Content** at http://links.lww.com/PQ9/A115 for Supplemental Table 2A)

**Fig. 1.**
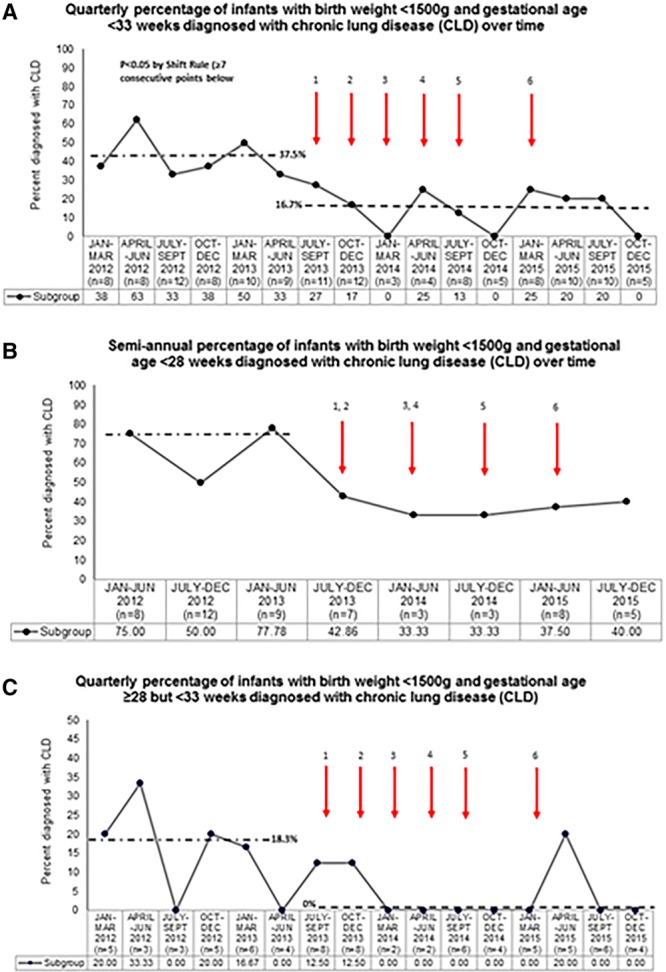
**A**, Quarterly percentage of infants with birth weights <1,500 g and GA younger than 33 weeks diagnosed with CLD over time. Numbered arrows indicate specific plan-do-study-act cycles, as follows: 1. Monthly task force meetings began, changed to exclusive bCPAP, implemented DR bCPAP, changed from nasogastric to orogastric tubes; 2. implemented trial off bCPAP guidelines, staff in-servicing on bCPAP interface, finalized guidelines; 3. staff in-servicing on guidelines; 4. encouraged guidelines for <28 weeks GA infants based on interim analysis, laminated guidelines for bedside posting; 5. revised extubation guidelines to add options of extubating to CPAP 7–9 cmH_2_O; allowed routine RAM cannula use at older than 34 weeks corrected age; 6. focused task force on <28 weeks GA infants, built portable bCPAP units, added option of INSURE technique of giving surfactant. **B**, semi-annual percentage of infants with birth weights younger than 28 weeks GA diagnosed with CLD over time. **C**, Quarterly percentage of infants with birth weights 28 weeks and older GA diagnosed with CLD over time.

The annotated Figure 1A indicates the timing and description of each set of our interventions. Compliance with each of the 5 elements of respiratory care, when applicable, were as follows: use of only bCPAP (82.4%, n = 78), DR CPAP (86.0%, n = 57), intubation criteria (94.7%, n = 75), extubation criteria (69.7%, n = 33), and weaning off CPAP (77.0%, n = 74).

Analysis of outcome measures using standard statistics is summarized in **Supplemental Digital content** for Table 2B, available at http://links.lww.com/PQ9/A115. All of the outcome measures had statistically significant improvements, except age at first extubation, percent extubation success, and CLD among infants born 28 weeks and older GA. Subsequent analysis was done of data over time, with all data points representing quarterly averages. The quarterly percentage of infants diagnosed with CLD decreased by 55.5%, from a median of 37.5% before to 16.7% after task force interventions (Fig. 1A). When we restricted the data to infants younger than 28 weeks GA, there were not enough data points to demonstrate if a significant reduction was achieved, with only 5 data points below the median (Fig. 1B). When the data were restricted to infants 28 weeks and older GA, CLD decreased from 18.3% before to a median of 0% after (Fig. 1C).

**Fig. 2.**
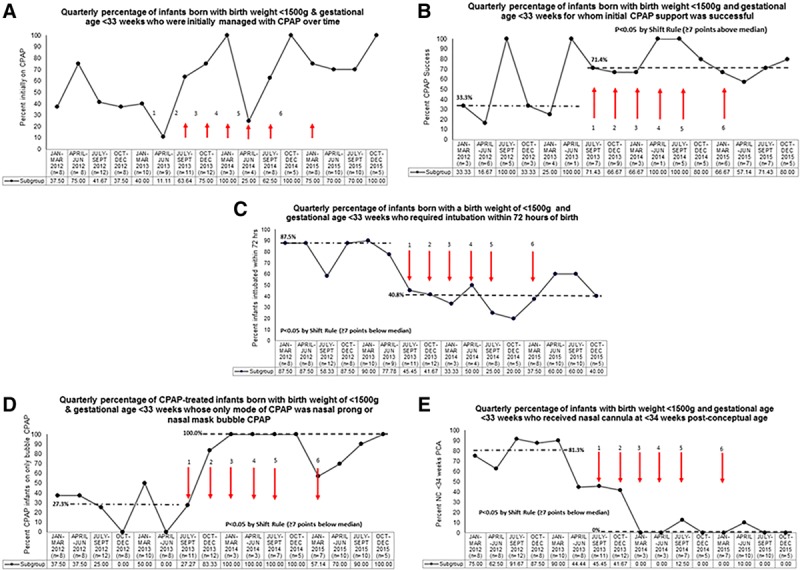
**A**, Quarterly percentage of infants born at <1,500 g birth weight and younger than 33 weeks gestation who were initially managed with CPAP, over time. Numbered arrows indicate specific plan-do-study-act cycles, as described in Figure 1. **B**, Quarterly percentage of infants born at <1,500 g birth weight and younger than 33 weeks gestation for whom initial CPAP was successful, over time. Denominator for this metric is the number of infants born each quarter who were initially managed with CPAP. **C**, Quarterly percentage of infants born at <1,500 g birth weight and younger than 33 weeks gestation who required intubation within 72 hours of birth. **D**, *Quarterly* percentage of infants born with birth weight <1,500 g GA younger than 33 weeks whose only mode of CPAP was nasal prong or nasal mask bubble CPAP, over time. Denominator for this metric is the number of infants born each quarter who were ever treated with CPAP. **E**, Quarterly percentage of infants born with birth weight <1,500 g GA younger than 33 weeks who received nasal cannula at younger than 34 weeks PCA, over time.

The quarterly percentage of infants initially managed with CPAP did not reach significance (Fig. 2A), but the percentage of infants for whom initial CPAP was successful increased from 33.3% to 71.4% (Fig. 2B). Fewer infants required intubation at <72 hours age; the median decreased from 87.5% to 40.8%. The percentage of CPAP-treated infants whose only mode of CPAP was nasal prong or nasal mask bCPAP increased from a median of 27.3% to 100.0%. The quarterly percentage of infants who received nasal cannula at younger than 34 weeks PCA decreased from 68.8% to 27.1% (Fig. 2C–E). Quarterly average days of CPAP significantly increased over time, and the average days of MV or oxygen significantly declined (see **Supplemental Digital Content**, available at http://links.lww.com/PQ9/A130 for Supplemental Figure 2A–C). There were no differences in the average hours at first extubation or the quarterly percentage of infants successfully extubated (data not shown) or in the percentage of infants receiving caffeine (93% before, 95% after, *P* = 0.64.)

There were no differences in pneumothorax, any ROP, or use of postnatal steroids, but there was a significant decrease in the incidence of PDA, declining from 60% before to 33% after (*P* < 0.01, see **Supplemental Digital Content** for Supplemental Table 2B) despite similar use of echocardiogram during the first 2 weeks of life (73% before, 63% after, *P* = 0.25). In subgroup analysis, we found the reduction in PDA was predominantly seen among infants born at 28 weeks and older GA, approaching significance at *P* = 0.05, but was not significant among those born at younger than 28 weeks GA. The average length of stay declined somewhat from 96.7 to 88.2 days, but this did not reach statistical significance (see **Supplemental Digital Content** for Supplemental Table 2B).

## DISCUSSION

We implemented a bundle of respiratory care practices and optimized CPAP delivery and reached our goal of reducing the incidence of CLD among VLBW infants born before 33 weeks GA at BMC by ≥50%. Our measures confirmed major changes in respiratory care practices. We changed our preferred method of CPAP from one of several options to nearly exclusive bCPAP. We demonstrated an increase in CPAP success among infants initially managed on CPAP, with fewer infants intubated within the first 72 hours of life. Compliance with the new respiratory care practices was generally high. These changes in management did not result in any increase in mortality or measured adverse outcomes. The incidence of PDA was significantly lower after our interventions without any co-incident change in surveillance or treatment. The novelty of this project was in the comprehensive change in management from birth through 34 weeks PCA, the emphasis on accountability by real-time monitoring of compliance, and use of a previously successful approach in a disparate NICU with similar results.

We outlined the rationale for our respiratory interventions in a prior publication,^13^ and since this publication, there were additional studies to support weaning off CPAP directly to RA rather than first to nasal cannula.^17–20^ The success of this overall approach rests heavily upon the ability to provide effective CPAP. CPAP failure is more likely if: the interface fits poorly; the mouth is frequently open (resulting in pressure and flow leakage); the stomach is poorly ventilated (resulting in excessive abdominal distention), or the interface causes skin breakdown that results in the abandonment of CPAP in favor of another mode of support. In-servicing the staff to ensure proper sizing and application of the CPAP interface was critical. Changing from nasogastric to orogastric tubes was done to maximize nasal patency for air movement and to allow for larger diameter (≥6.5F) gavage tubes for venting the stomach. Use of chin straps, while initially unpopular, facilitated proper delivery of CPAP pressure and flow. Ensuring the adoption of these practices required frequent monitoring and reminders to the staff, but adoption improved over time.

As expected, there was a range of enthusiasm among the staff with making such large changes in management. Several team components were essential for our success: a dedicated and visible leader, a strong multidisciplinary team, and steadfast support from NICU leadership. Other important elements included creating a sense of urgency by noting BMC’s high baseline rate of CLD; discussing the consequences of CLD; inviting everyone to participate in the task force; communicating management changes widely; and sharing results during meetings, on bulletin boards, via occasional email blasts, and during personal communications.

Having at least one identified person to monitor compliance is helpful. It is important to recognize that while unanimous enthusiasm for change among staff members may be ideal, it is generally not possible to achieve, and the goal should be to achieve compliance. While we did not specifically measure staff satisfaction, we tried to acknowledge the positive efforts of many and address all complaints.

The respiratory care bundle was nearly identical to that successfully implemented at SEMC, and the results were similar despite significant differences between institutions.^13^ The clinical team at SEMC consisted of attending neonatologists, experienced neonatal nurse practitioners, and Neonatal-Pediatric Specialist-certified RTs who covered the NICU exclusively and were present at all high-risk deliveries. While technically a teaching hospital, the neonatal care at SEMC was not provided by any trainees. The NICU at SEMC was smaller, and the population was gestationally more mature, predominantly white, middle class, and privately insured.

In contrast, while an attending neonatologist leads the clinical team at BMC, all direct medical care is provided by rotating pediatric residents, not experienced nurse practitioners. Few of the RTs are Neonatal-Pediatric Specialist-certified, most are predominantly adult therapists who are simultaneously assigned to other hospital areas in addition to the NICU, and they id not attend deliveries unless requested. The population at BMC is predominantly African American and/or Hispanic, with a high percentage of inner-city low-income patients, and Medicaid insures nearly all patients. Our cohort was gestationally less mature, had a lower mean birth weight, and lower 5-minute Apgar scores than the cohort at SEMC. We believe that the similarity in results between SEMC and BMC speaks to the robustness of the interventions, as well as their adaptability to different clinical environments.

This report has several limitations that may affect the ability to reproduce such a significant reduction in CLD. First, we started with a high median quarterly incidence of CLD of 37.5%. For perspective, the median VON hospital rate for CLD among VLBW infants born at younger than 33 weeks GA from 2012 to 2015 was 21.3%, with an interquartile range of 12.4%–30.9%.^21^ While we achieved a large reduction, our new median quarterly incidence of CLD of 16.7% is still within the VON interquartile range.

Second, the lower average GA in the group born before the interventions compared with those born after may have influenced the results. We tried to account for this difference by separately analyzing the impact of our interventions on those born at younger than 28 weeks GA and those born at 28 weeks and older GA. The incidence of CLD is inversely related to GA, but our small sample size and project design do not favor further analysis of conclusions. A randomized clinical trial would be required to fully investigate the possibility of a differential effect of our interventions based on GA.

Third, CLD is a multifactorial disease. Exposure to MV and sO2 are 2 important and modifiable factors, but they are not the only factors. Respiratory practices vary between centers, so the impact of these interventions on any given NICU would be dependent on the difference between their current practices and those we implemented.

It is possible that other co-incident changes, interventions, and/or comorbidities could have affected the achieved improvement. There were no changes in practice regarding oxygen management at 36 weeks gestation. Interventions during the same time-period included a change in degree and duration of incubator humidity for VLBW infants, the new availability of starter parenteral nutrition, and provision of an enteral liquid protein supplement. Fluid management and nutrition are also important factors in the development of CLD. Our high incidence of necrotizing enterocolitis may have blunted the impact of our interventions. Further improvement may depend on our ability to reduce this complication, as the resulting inflammation increases the risk of CLD.

We collected data on other complications of prematurity to monitor for possible adverse effects of our interventions. The finding of reduced incidence of PDA was not anticipated. This outcome could also be affected by the pre-post difference in average GA, as the incidence of PDA is inversely related to GA. On sub-analysis of PDA by GA, we found that PDA was only less frequent among those born at 28 weeks and older. Again, an RCT would be better suited to address the differential effects of these interventions on PDA based on GA.

In summary, we reduced the incidence of CLD among VLBW infants born before 33 weeks GA at BMC by over 50% by implementing a bundle of specific respiratory care practices and optimizing CPAP delivery. Our process measures confirmed significant changes in respiratory management without an increase in mortality or any measured adverse outcome. The incidence of PDA was significantly lower after our interventions.

## ACKNOWLEDGMENTS

The authors wish to recognize the contributions and support of the additional members of the BMC CLD Taskforce, including Caitlin Mann-Bradley, RN, NNP, Daniel Gavin, RT, Elisha Wachman, MD, Margaret Parker, MD, Alice Wang, MD, Jane Nicotera, RN, Jessica Scola, RN, Phuong Vo, MD, MPH, and Tatiana Kiernan, PNP. This work was unfunded.

## DISCLOSURE

The authors have no financial interest to declare in relation to the content of this article.

## Supplementary Material
