# Supplementary material for: Improving Respiratory Support Practices to Reduce Chronic Lung Disease in Premature Infants
Source: Pediatr Qual Saf. 2019 Aug 9;4(4):e193. doi: 10.1097/pq9.0000000000000193 (PMC6708652; doi:10.1097/pq9.0000000000000193)
Supplement: Supplementary file 1 [file pqs-4-e193-s001.pdf]

Table 1, SDC: Comparison of Hospital, NICU, and Cohort Population Characteristics

<table>
<tr><td></td><td colspan="2">SEMC</td><td>BMC</td></tr>
<tr><td>Hospital Type</td><td colspan="2">Private, For-Profit Hospital</td><td>Private, Not-for-Profit
Safety-Net Hospital</td></tr>
<tr><td>Licensed Beds</td><td colspan="2">267</td><td>567</td></tr>
<tr><td>Annual Births (2016)</td><td colspan="2">971</td><td>2810</td></tr>
<tr><td>NICU Beds</td><td colspan="2">18</td><td>21</td></tr>
<tr><td>Characteristics of Hospital Neighborhood</td><td colspan="2">Urbanized, Residential</td><td>Urban, Inner City</td></tr>
<tr><td>Teaching Designation</td><td colspan="2">Teaching Affiliate</td><td>Academic Medical Center</td></tr>
<tr><td>Interns and/or Resident Coverage in NICU</td><td colspan="2">No</td><td>Yes</td></tr>
<tr><td>NICU Staffing</td><td colspan="2">Attending Neonatologist
Neonatal Nurse Practitioner
Respiratory Therapist (RT)
NICU Nurse</td><td>Attending Neonatologist
Pediatric Resident
RT available in hospital
NICU Nurse</td></tr>
<tr><td>Demographics of Infants Included in each Initiative</td><td>SEMC*</td><td>BMC</td><td>P-value</td></tr>
<tr><td>Cohort Size</td><td>121</td><td>131</td><td>N/A</td></tr>
<tr><td>Gestational Age, mean ± SD</td><td>30.1 ± 2</td><td>28.4 ± 2.3</td><td>< 0.01</td></tr>
<tr><td>Birth Weight, mean ± SD</td><td>1393.5 ± 400</td><td>1034.9 ± 288</td><td>< 0.01</td></tr>
<tr><td>Small for Gestational Age, (% of cohort)</td><td>12 (9.9%)</td><td>18 (13.7%)</td><td>0.35</td></tr>
<tr><td>Nonwhite race (% of cohort)</td><td>54 (45%)</td><td>117 (89%)</td><td>< 0.01</td></tr>
<tr><td>Cesarean Delivery</td><td>88 (72.7%)</td><td>96 (73.3%)</td><td>0.91</td></tr>
<tr><td>5-min Apgar score, mean ± SD</td><td>8.3 ± 1.0</td><td>6.9 ± 2.3</td><td>< 0.01</td></tr>
<tr><td>Male Gender</td><td>70 (58%)</td><td>57 (44%)</td><td>0.03</td></tr>
<tr><td colspan="4">*Data from published initiative[13]</td></tr>
</table>

Table 1, SDC

Comparison of hospital, NICU and cohort populations at St. Elizabeth's Medical Center compared to Boston Medical Center.
