# Supplementary material for: Improving Respiratory Support Practices to Reduce Chronic Lung Disease in Premature Infants
Source: Pediatr Qual Saf. 2019 Aug 9;4(4):e193. doi: 10.1097/pq9.0000000000000193 (PMC6708652; doi:10.1097/pq9.0000000000000193)
Supplement: Supplementary file 2 [file pqs-4-e193-s002.pdf]

Table 2A, SDC: Demographic Characteristics

	Total Cohort		Pre-Taskforce		Post-Taskforce		
N	131		55		76		
Continuous Variables	Mean	SD	Mean	SD	Mean	SD	P-Value
Gestational age, weeks	28.4	2.3	27.9	2.1	28.8	2.4	0.03
Birth weight, g	1034.9	288.1	991.2	263.8	1066.6	302.3	0.14
Categorical Variables	N	Percent	N	Percent	N	Percent	
Sex							
Male	57	44%	24	44%	33	43%	0.99
Maternal race/ethnicity							
African American	68	52%	24	44%	44	58%	0.15
White	14	11%	5	9%	9	12%	0.62
Hispanic	40	31%	21	38%	19	25%	0.11
Any antenatal steroids	114	87%	47	85%	67	88%	0.65

Table 2B, SDC: Outcome and Balancing Measures

	Pre-Taskforce		Post-Taskforce		
Outcome Measures		N		N	P-Value
Initial management with CPAP	40.0%	55	69.7%	76	< 0.01
Initial CPAP successful	45.5%	22	71.7%	53	0.03
Intubation 1st 72 hours of life	80.0%	55	42.1%	76	0.01
Age at 1st extubation, hours (mean ± SD)	177.1 ± 308.0	43	171.0 ± 302.9	33	0.93
1st Extubation successful	70.5%	44	56.3%	32	0.21
Bubble CPAP only form of CPAP used	18.5%	54	81.9%	72	< 0.01
Nasal cannula <34 weeks post-conceptual age	76.4%	55	15.8%	76	< 0.01
Days on CPAP (mean ± SD)	19.4 ± 17.1	54	30.0 ± 26.4	76	0.01
Days on mechanical ventilation (mean ± SD)	11.1 ± 16.0	54	6.22 ± 12.0	76	0.02
Days on supplemental oxygen (mean ± SD)	44.0 ± 40.2	53	25.6 ± 42.3	76	0.01
Chronic lung disease VLBW infants <33 weeks GA	41.8%	55	17.1%	76	0.01
Chronic lung disease VLBW infants <28 weeks GA	65.5%	29	38.5%	26	0.04
Chronic lung disease VLBW infants ≥28 weeks GA	15.4%	26	6.0%	50	0.18
Balancing Measures					
Pneumothorax	4%	55	1%	76	0.37
Patent ductus arteriosus VLBW infants <33 weeks GA	60%	55	33%	76	< 0.01
Patent ductus arteriosus VLBW infants <28 weeks GA	79%	29	62%	26	0.15
Patent ductus arteriosus VLBW infants ≥28 weeks GA	38%	26	18%	50	0.05
Any retinopathy of prematurity	56%	55	42%	76	0.11
Post-natal steroids for CLD	20%	55	16%	76	0.53
Length of stay, days (mean ± SD)	96.7 ± 45.3	54	88.2 ± 52.8	76	0.17

Table 2, SDC

A, Demographic characteristics for infants with birth weight <1500 grams and GA <33 weeks born before and after the chronic lung disease (CLD) task force interventions. B, Outcome and balancing measures analyzed using standard statistics comparing infants with birth weight <1500 grams and GA <33 weeks born before and after the CLD task force interventions.
