# Supplementary material for: Improving Respiratory Support Practices to Reduce Chronic Lung Disease in Premature Infants
Source: Pediatr Qual Saf. 2019 Aug 9;4(4):e193. doi: 10.1097/pq9.0000000000000193 (PMC6708652; doi:10.1097/pq9.0000000000000193)
Supplement: Supplementary file 3 [file pqs-4-e193-s003.pptx]

## Slide 1
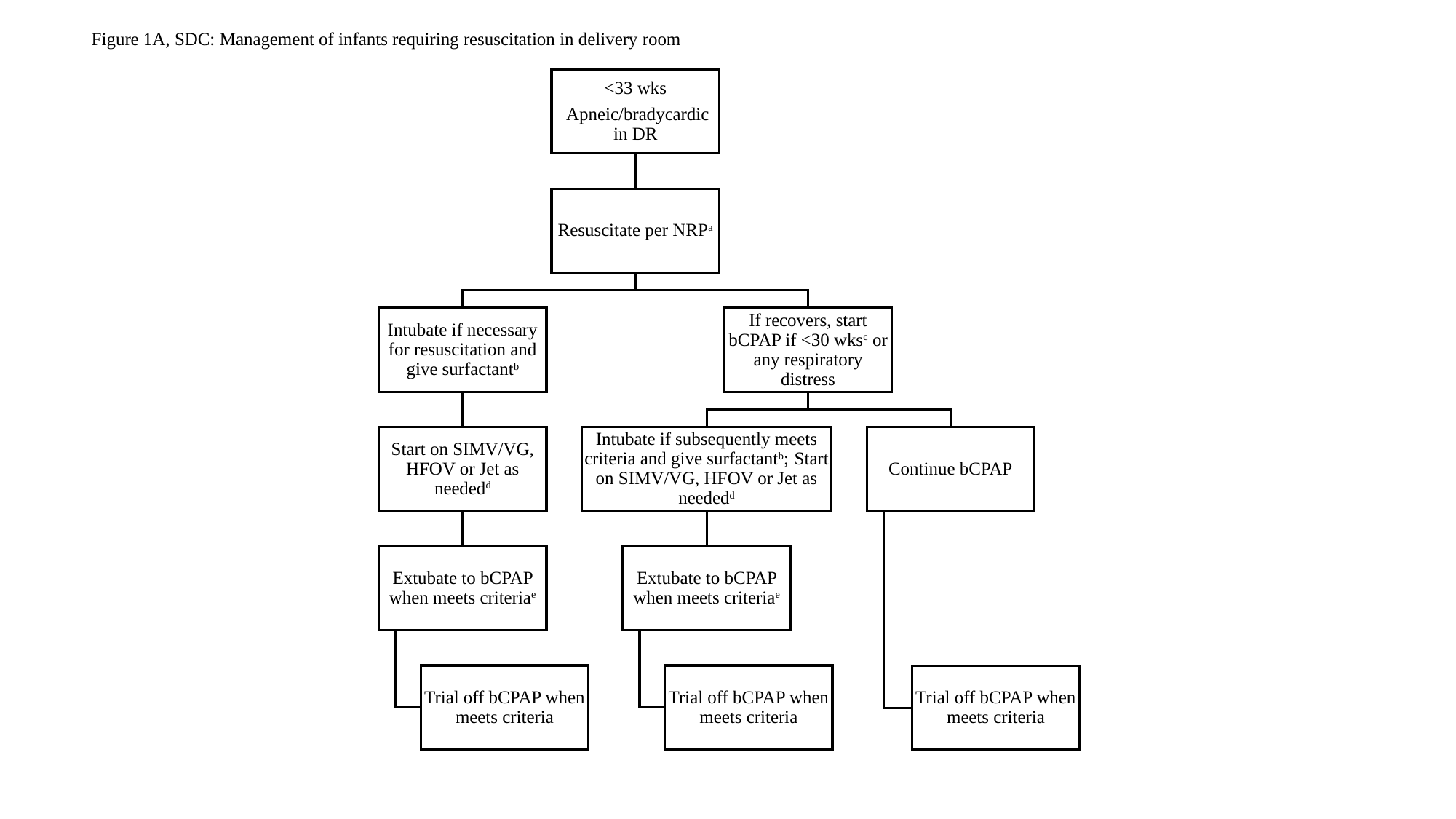

Figure 1A, SDC: Management of infants requiring resuscitation in delivery room

## Slide 2
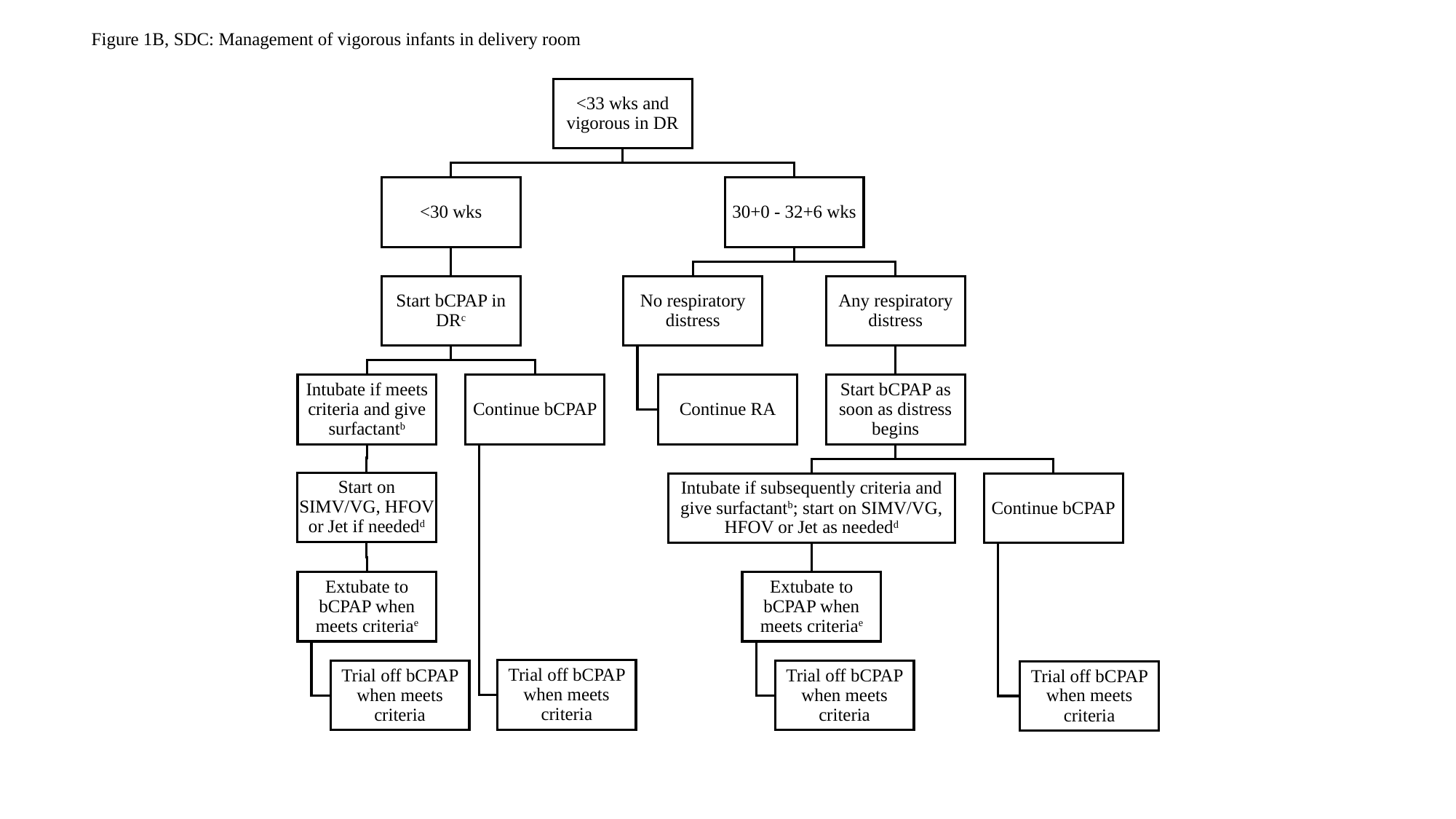

Figure 1B, SDC: Management of vigorous infants in delivery room

## Slide 3
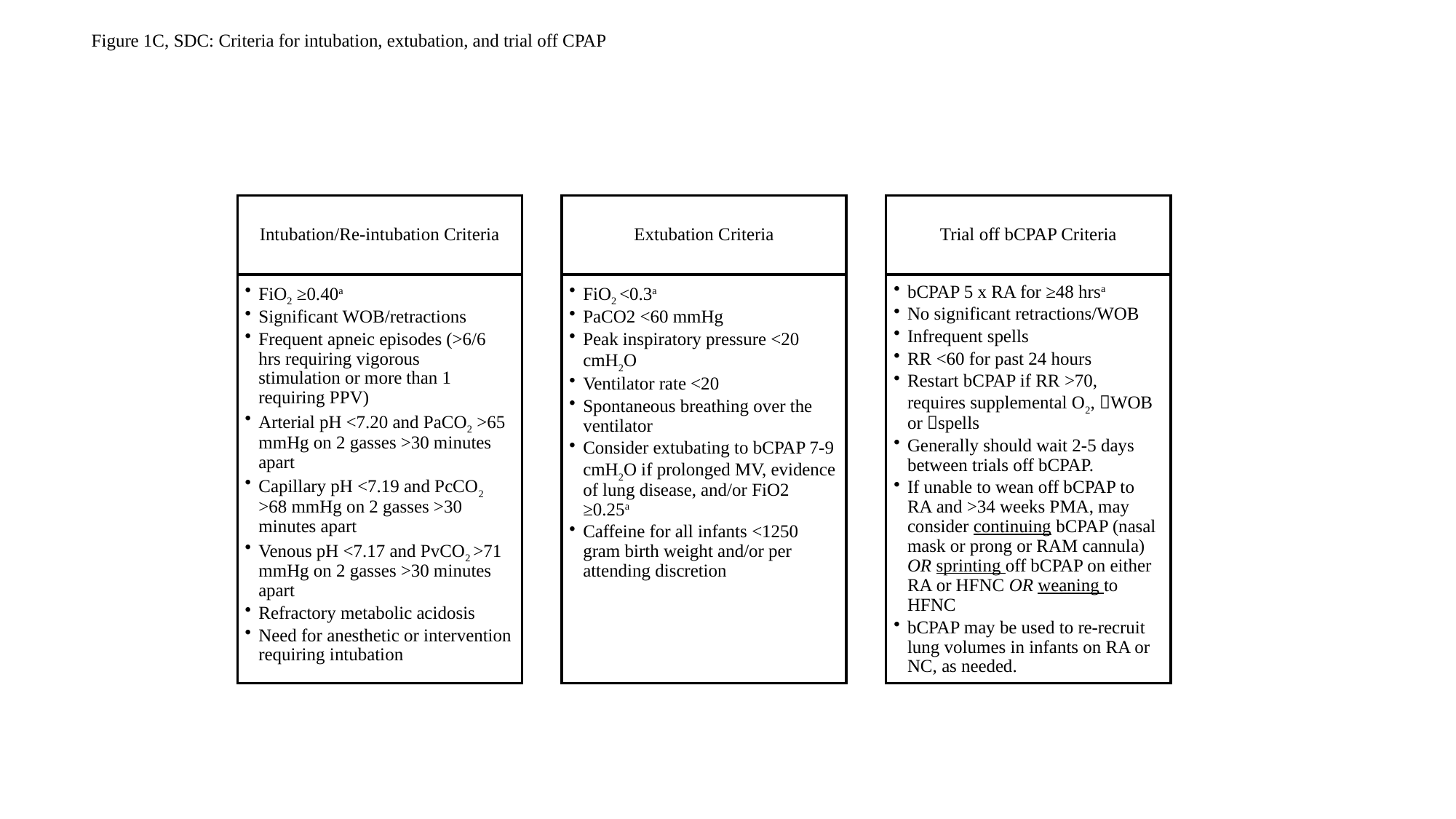

Figure 1C, SDC: Criteria for intubation, extubation, and trial off CPAP
