# Supplementary material for: Improving Respiratory Support Practices to Reduce Chronic Lung Disease in Premature Infants
Source: Pediatr Qual Saf. 2019 Aug 9;4(4):e193. doi: 10.1097/pq9.0000000000000193 (PMC6708652; doi:10.1097/pq9.0000000000000193)
Supplement: Supplementary file 4 [file pqs-4-e193-s004.pptx]

## Slide 1
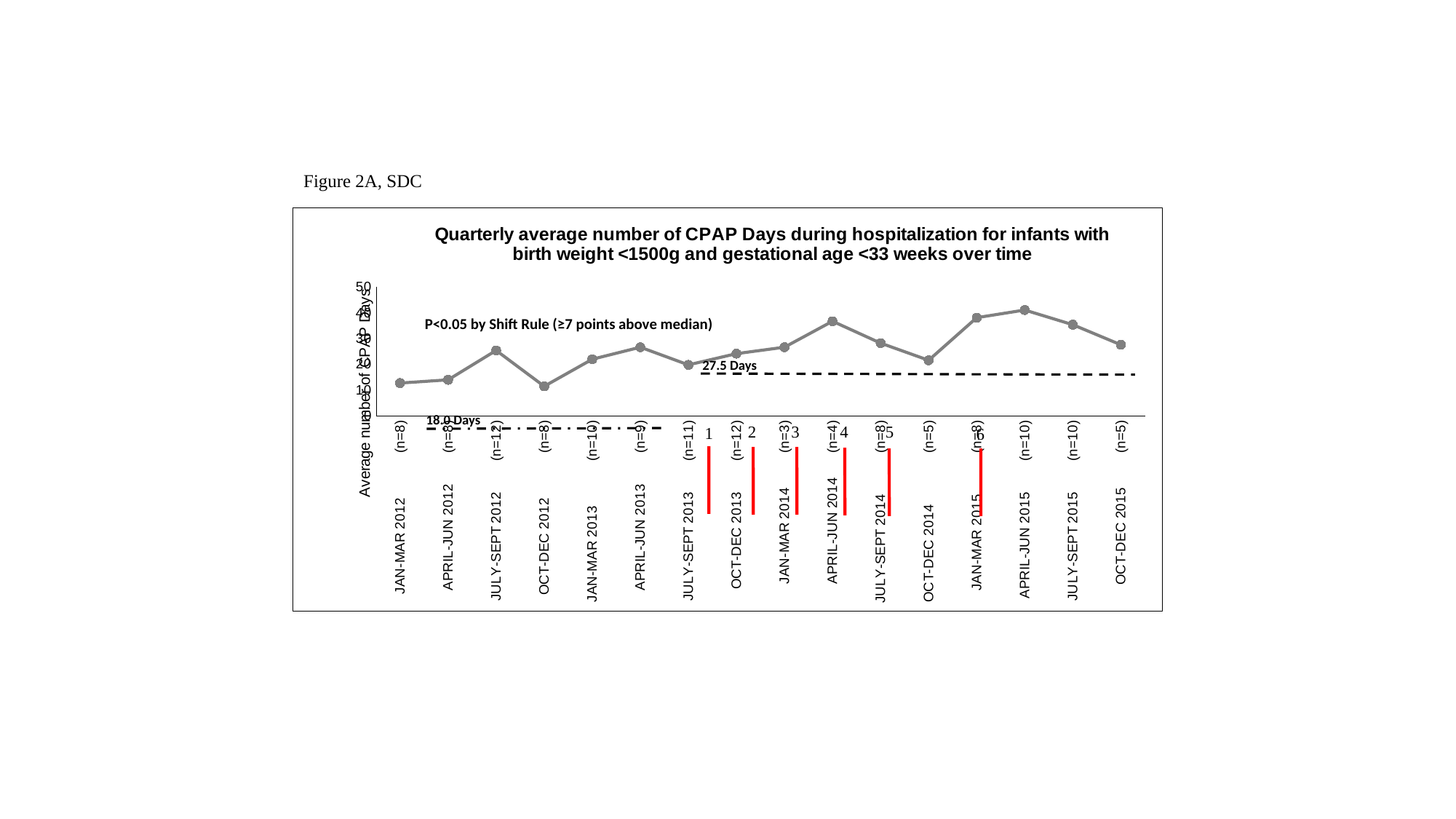

Figure 2A, SDC
### Chart: Quarterly average number of CPAP Days during hospitalization for infants with birth weight <1500g and gestational age <33 weeks over time
| Category | |
|---|---|
| JAN-MAR 2012 (n=8) | 12.75 |
| APRIL-JUN 2012 (n=8) | 14.0 |
| JULY-SEPT 2012 (n=12) | 25.416666666666668 |
| OCT-DEC 2012 (n=8) | 11.5 |
| JAN-MAR 2013 (n=10) | 22.0 |
| APRIL-JUN 2013 (n=9) | 26.666666666666668 |
| JULY-SEPT 2013 (n=11) | 19.818181818181817 |
| OCT-DEC 2013 (n=12) | 24.166666666666668 |
| JAN-MAR 2014 (n=3) | 26.666666666666668 |
| APRIL-JUN 2014 (n=4) | 36.75 |
| JULY-SEPT 2014 (n=8) | 28.25 |
| OCT-DEC 2014 (n=5) | 21.6 |
| JAN-MAR 2015 (n=8) | 38.125 |
| APRIL-JUN 2015 (n=10) | 41.1 |
| JULY-SEPT 2015 (n=10) | 35.4 |
| OCT-DEC 2015 (n=5) | 27.6 |P<0.05 by Shift Rule (≥7 points above median)
27.5 Days
18.0 Days

## Slide 2
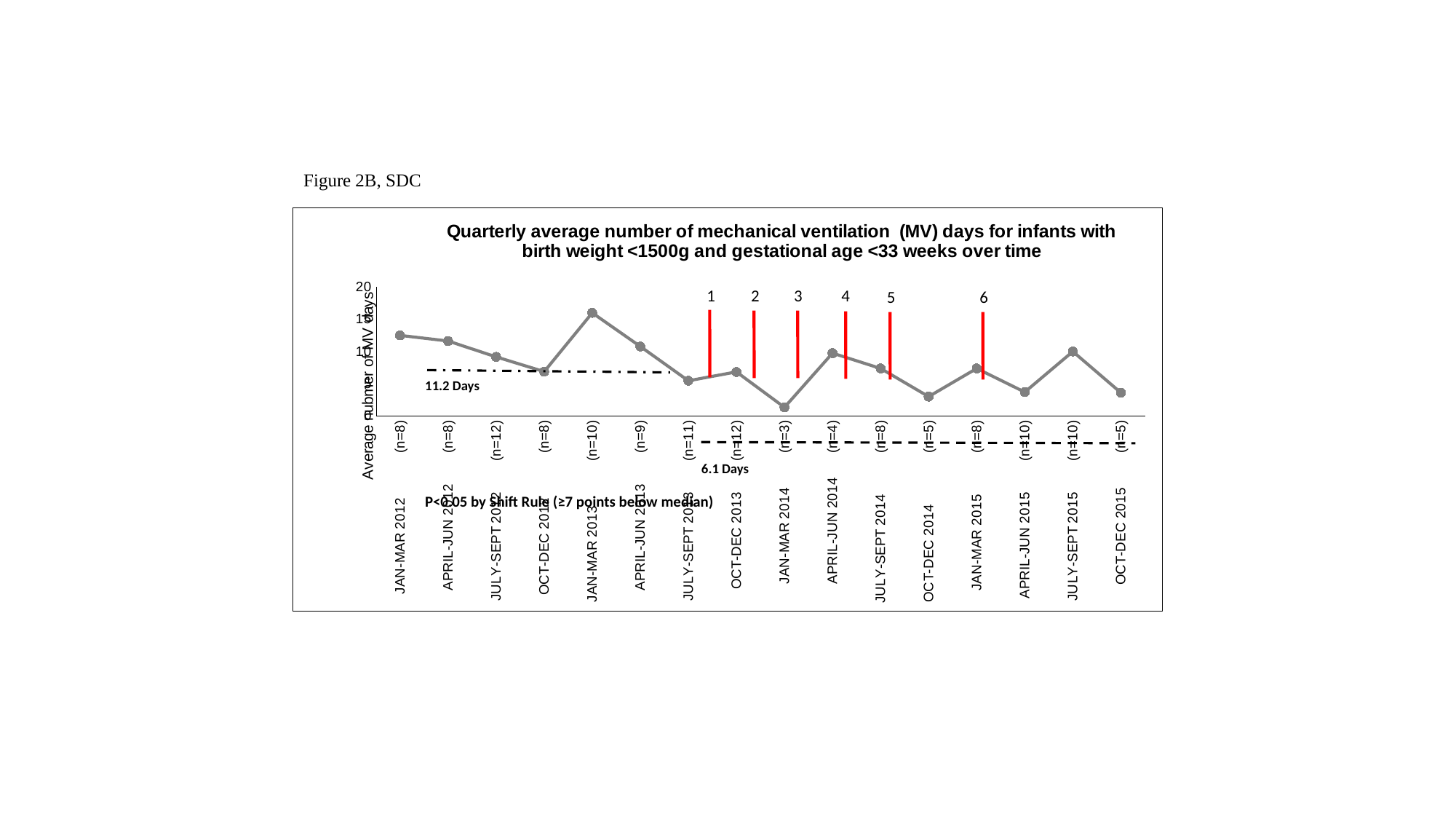

Figure 2B, SDC
### Chart: Quarterly average number of mechanical ventilation (MV) days for infants with birth weight <1500g and gestational age <33 weeks over time
| Category | |
|---|---|
| JAN-MAR 2012 (n=8) | 12.5 |
| APRIL-JUN 2012 (n=8) | 11.625 |
| JULY-SEPT 2012 (n=12) | 9.166666666666666 |
| OCT-DEC 2012 (n=8) | 6.875 |
| JAN-MAR 2013 (n=10) | 16.0 |
| APRIL-JUN 2013 (n=9) | 10.777777777777779 |
| JULY-SEPT 2013 (n=11) | 5.454545454545454 |
| OCT-DEC 2013 (n=12) | 6.833333333333333 |
| JAN-MAR 2014 (n=3) | 1.3333333333333333 |
| APRIL-JUN 2014 (n=4) | 9.75 |
| JULY-SEPT 2014 (n=8) | 7.375 |
| OCT-DEC 2014 (n=5) | 3.0 |
| JAN-MAR 2015 (n=8) | 7.375 |
| APRIL-JUN 2015 (n=10) | 3.7 |
| JULY-SEPT 2015 (n=10) | 10.0 |
| OCT-DEC 2015 (n=5) | 3.6 |1
2
3
4
5
6
11.2 Days
6.1 Days
P<0.05 by Shift Rule (≥7 points below median)

## Slide 3
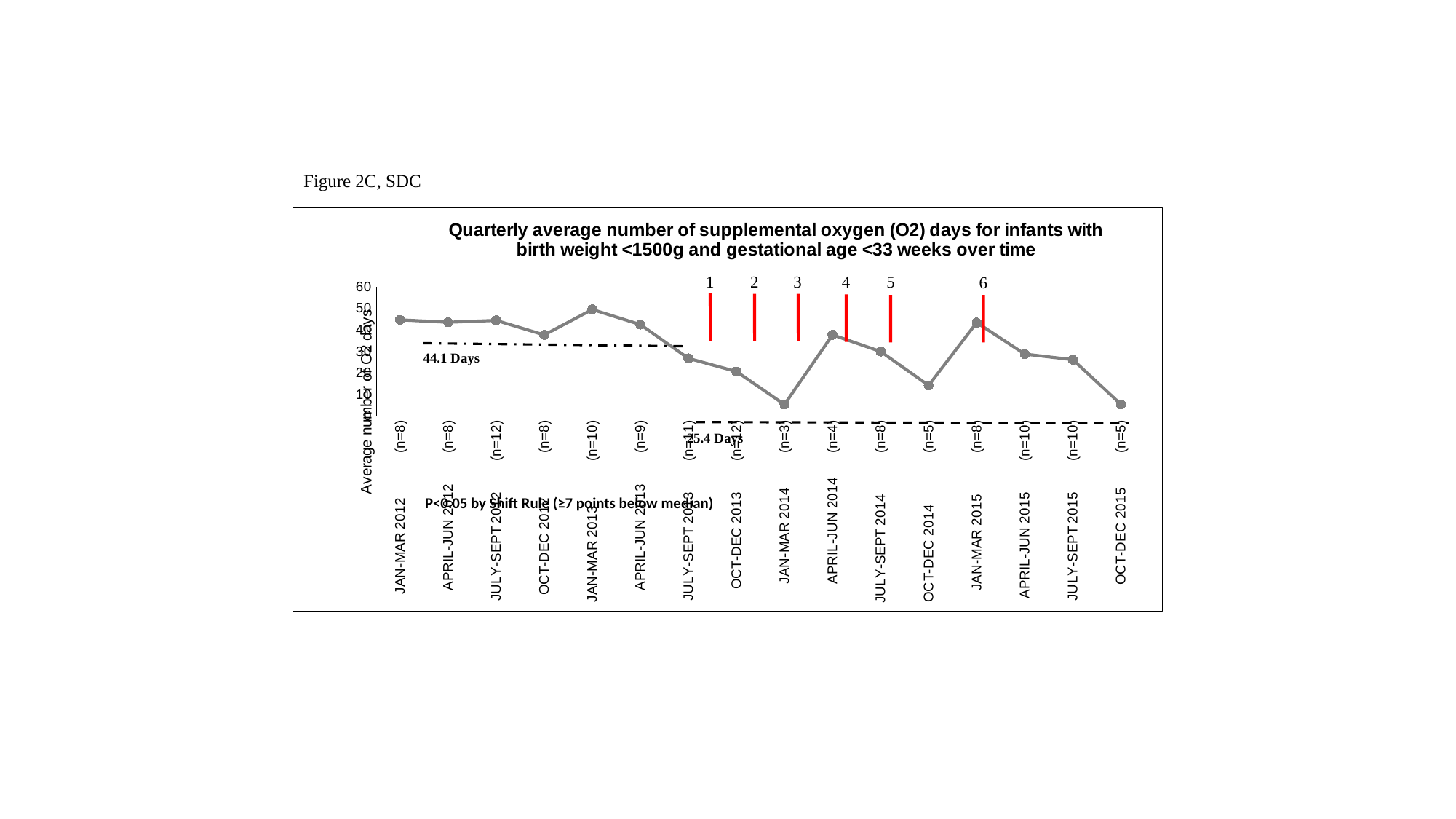

Figure 2C, SDC
### Chart: Quarterly average number of supplemental oxygen (O2) days for infants with birth weight <1500g and gestational age <33 weeks over time
| Category | |
|---|---|
| JAN-MAR 2012 (n=8) | 44.75 |
| APRIL-JUN 2012 (n=8) | 43.625 |
| JULY-SEPT 2012 (n=12) | 44.5 |
| OCT-DEC 2012 (n=8) | 37.714285714285715 |
| JAN-MAR 2013 (n=10) | 49.55555555555556 |
| APRIL-JUN 2013 (n=9) | 42.55555555555556 |
| JULY-SEPT 2013 (n=11) | 26.818181818181817 |
| OCT-DEC 2013 (n=12) | 20.666666666666668 |
| JAN-MAR 2014 (n=3) | 5.333333333333333 |
| APRIL-JUN 2014 (n=4) | 37.75 |
| JULY-SEPT 2014 (n=8) | 30.0 |
| OCT-DEC 2014 (n=5) | 14.2 |
| JAN-MAR 2015 (n=8) | 43.5 |
| APRIL-JUN 2015 (n=10) | 28.8 |
| JULY-SEPT 2015 (n=10) | 26.2 |
| OCT-DEC 2015 (n=5) | 5.4 |P<0.05 by Shift Rule (≥7 points below median)
